# Abdominal Surgery

**Published:** 1894-01-06

**Authors:** 


### ABDOMINAL SURGERY.

The regional sub-division of the abdomen has never been laid down with sufficient uniformity by the various text-books of anatomy to establish a definite understanding of such words as epigastric, lumbar, &c., and Mr. Anderson,[1] in introducing a discussion on this subject, thought that a more accurate system of delimitation could be obtained by converting the lines of segmentation into planes which passed through the trunk, and were fixed as to position by well-defined bony landmarks. Perhaps the best of these more exact topographical methods is that of Professor Cunningham, who proposes to divide the abdominal cavity into sections by means of two transverse and two longitudinal planes. The upper transverse plane corresponds to the level of the lowest point of the costal border, as seen from the front (*i.e.*, the cartilage of the tenth rib), and passes through the disc between the second and third lumbar vertebræ, while the lower plane passes through the well-known tubercle at the outer border of the iliac crest, coinciding posteriorly with the level of the fifth lumbar vertebra. The longitudinal plane corresponds to that commonly in use, and extends vertically upwards (or parallel to the median plane) from the middle of Poupart's ligament.

On the subject of abdominal operations generally, Mr. Morison claims[2] that the general principles of surgery must be carried out in these as in other operations, and that the causes of failure in abdominal surgery are those common to all major operations, the chief being: 1. Septic contamination. 2. Imperfect arrest of hæmorrhage. 3. Neglect of shock. He speaks highly of Mr. Tait's method of treatment of "after hæmorrhage" appearing through an abdominal drainage tube: "The tube is pumped dry, and a syringeful of perchloride of iron solution slowly injected (a piece of solid perchloride of iron the size of a filbert, dissolved in a tumbler of water, makes a solution of suitable strength). The solution is pumped in and out a few times till bleeding ceases.

In spite of the fact that iodoform apparently exercises no restraining influence on the growth of organisms outside the body, yet it is unquestionably true that it has a powerful antiseptic action on wounds—the most probable explanation of this paradoxical behaviour being that suggested by Behring, that iodoform produces its beneficial results not by acting directly on the bacteria, but by inducing chemical changes in their toxic products. Mr. Treves[3] has spoken favourably of the use of this drug in abdominal operations, and believes that in cases of nephrectomy, fæcal fistula, abscess of liver, and other cases in which he has employed it, he has obtained such good results that he believes iodoform played an important part in the healing process.

Mr. Tait's treatment of incipient peritonitis after abdominal operations by purgation is now well known and often practised, but Mr. Cant points out[4] that it is chiefly in that form of peritonitis which follows the stasis and congestion caused by the necessary injury to the peritoneum and viscera during the operation that this method of treatment is most valuable. If not relieved, this stasis soon passes on to congestion and inflammation, the incipient symptoms being abdominal distension and vomiting. For this the following plan is suggested :—

1. To give nothing by the mouth except a little warm water for the first thirty-six hours or longer, should there be any signs of vomiting.
2. To give no opium, for this tends to mask symptoms, increase the liver engorgement and diminish the peristaltic action of the intestines.
3. To relieve distension and to act on the bowels, by passing a long tube into the rectum occasionally, and by the administration of a turpentine enema, followed by three or four grains of calomel if necessary.

The treatment of thirst after abdominal operations by means of rectal injections of warm water is by no means new, but does not seem to be so generally known as it ought. Mr. Cathcart[5] has drawn attention to the usefulness of this method when for various reasons it is not desirable to administer much fluid by the mouth. About half a pint of warm water is injected, and this is repeated every two or three hours if necessary.

The bacillus coli communis—which has now been identified with the bacillus fœtidus of Passet, the bacillus pyogenes of Albarran and Clado—seems to be taking a very prominent position as an ætiological factor in abdominal diseases. A summary of Macaigne's views of this important organism is given by Dr. Roswell Park as follows[6]:

Ordinarily inoffensive, it may, from causes not yet ascertained, acquire a greater or lesser degree of virulence, according to which it may determine one or more of the following lesions: A. Infectious enteritis; (*a*) Acute form. 1. The algid forms: cholera nostras, cholera infantum. 2. The pyretic form: post-puerperal pseudo-infection, &c. (*b*) The chronic form. 1. The wasting enteritis of children and of adults. B. Dysentery. Then if the intestinal barrier is broken down we may have peritonitis and herniary cholera. Probably most, if not all, cases of appendicitis fall also under this head.

The ascent of the colon bacillus along the biliary passage may provoke, according to its virulence (1), Simple biliary infection; (2) acute jaundice; (3) suppurative angio-cholitis. In the alimentary canal it is capable of provoking—in the mouth, false membrane; in the stomach and intestines, infarcts; and lower down, peri-rectal abscesses. The bacillus penetrating the intestinal mucosa may infect parts or organs at a distance; for example, the endocardium, the thyroid body, the lungs, &c.

Finally, the bacillus may in some way, not yet fully understood, provoke meningitis, urinary infection, pulmonary and articular lesions.

[1] Brit. Med. Journ., 1893, vol. ii., p. 679. [2] Edin. Med. Journ., July, 1893, p. 22. [3] Lancet, 1893, vol. i., p. 1,375. [4] Sheffield Med. Journ., July, 1893. [5] Edin. Med. Journ., Sept., 1893, and Brit. Med. Journ., Oct. 14, 1893. [6] Annals of Surgery, Sept., 1893.

